# Supplementary figures and images for: Senescent response in inner annulus fibrosus cells in response to TNFα, H2O2, and TNFα-induced nucleus pulposus senescent secretome
Source: PLoS One. 2024 Jan 5;19(1):e0280101. doi: 10.1371/journal.pone.0280101 (PMC10769024; doi:10.1371/journal.pone.0280101)

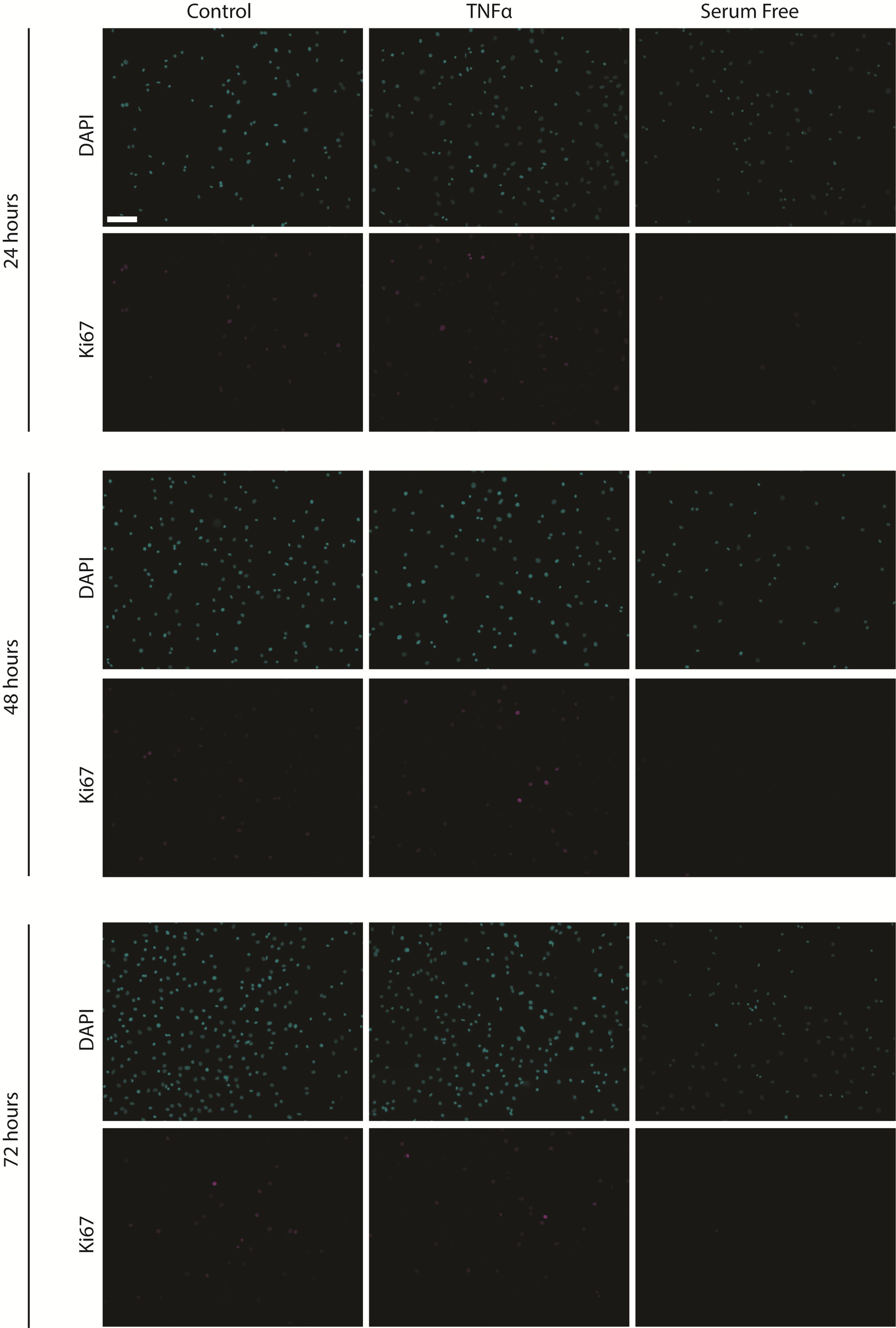

Supplement: S1 Fig — DAPI was used as a nuclear stain. Scale bar = 100 μm. (TIF) [file pone.0280101.s001.tif]

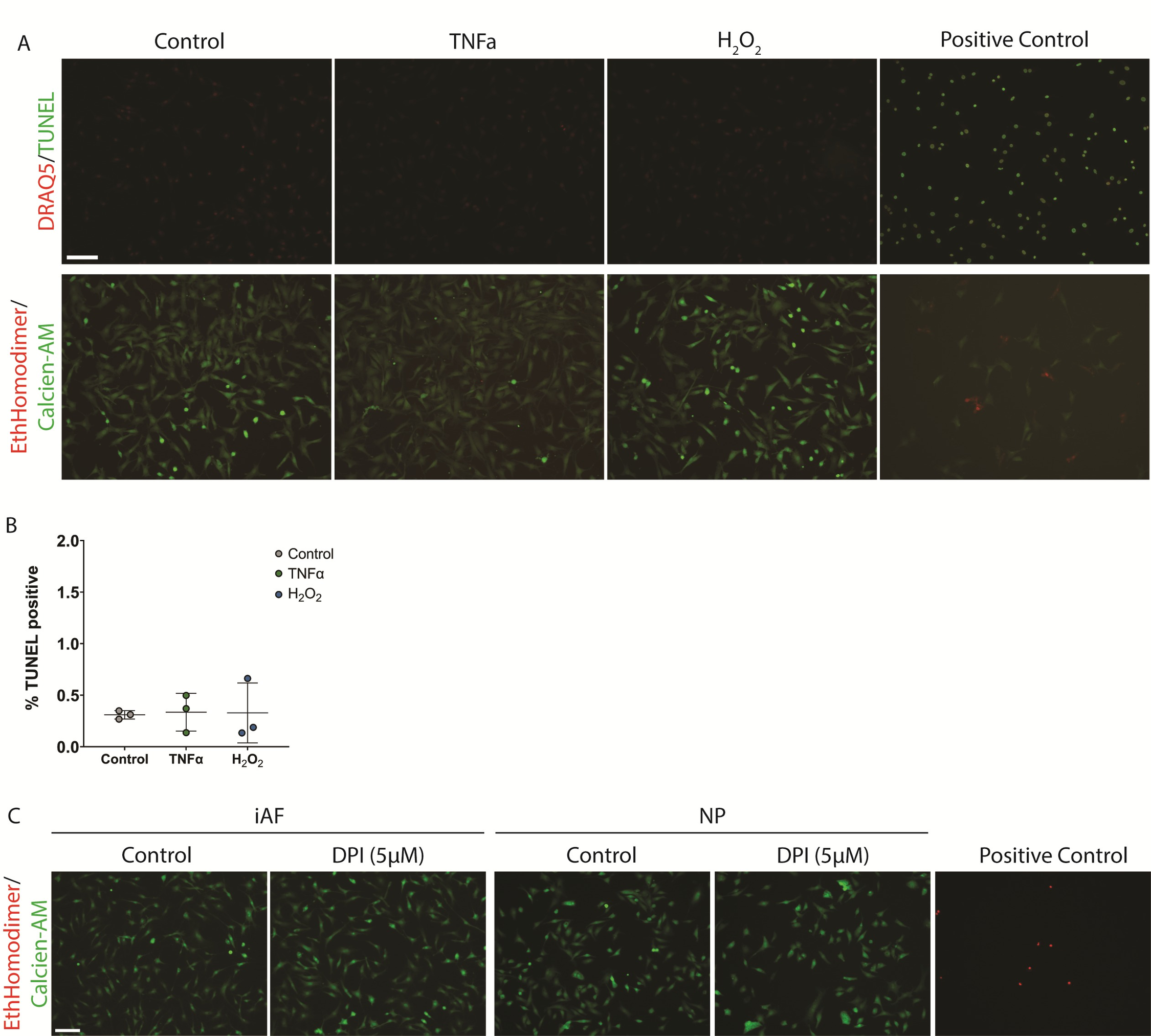

Supplement: S2 Fig — (A) Representative images of TUNEL staining with DRAQ5 as a nuclear stain and ethidium homodimer (red)/Calcein-AM (green) following exposure to TNFα (40 ng/mL) or H2O2 (50 μM). Positive controls were exposed to DNAse I for TUNEL staining and 50 mM H2O2 for ethidium homodimer/calcein-AM staining. (B) Quantification of percentage of TUNEL positive iAF cells exposed to TNFα and H2O2. (C) Representative images of ethidium homodimer (red)/Calcein-AM (green) stained iAF and NP cells following exposure to diphenyleneiodonium chloride (DPI) (5 μM). Student’s T-test was used for statistical analyses of DPI study, one-way ANOVA with Tukey’s post-hoc was used for iAF TUNEL and iAF live-dead quantification. N = 3 for all experiments. Scale bars = 100 μm. (TIF) [file pone.0280101.s002.tif]

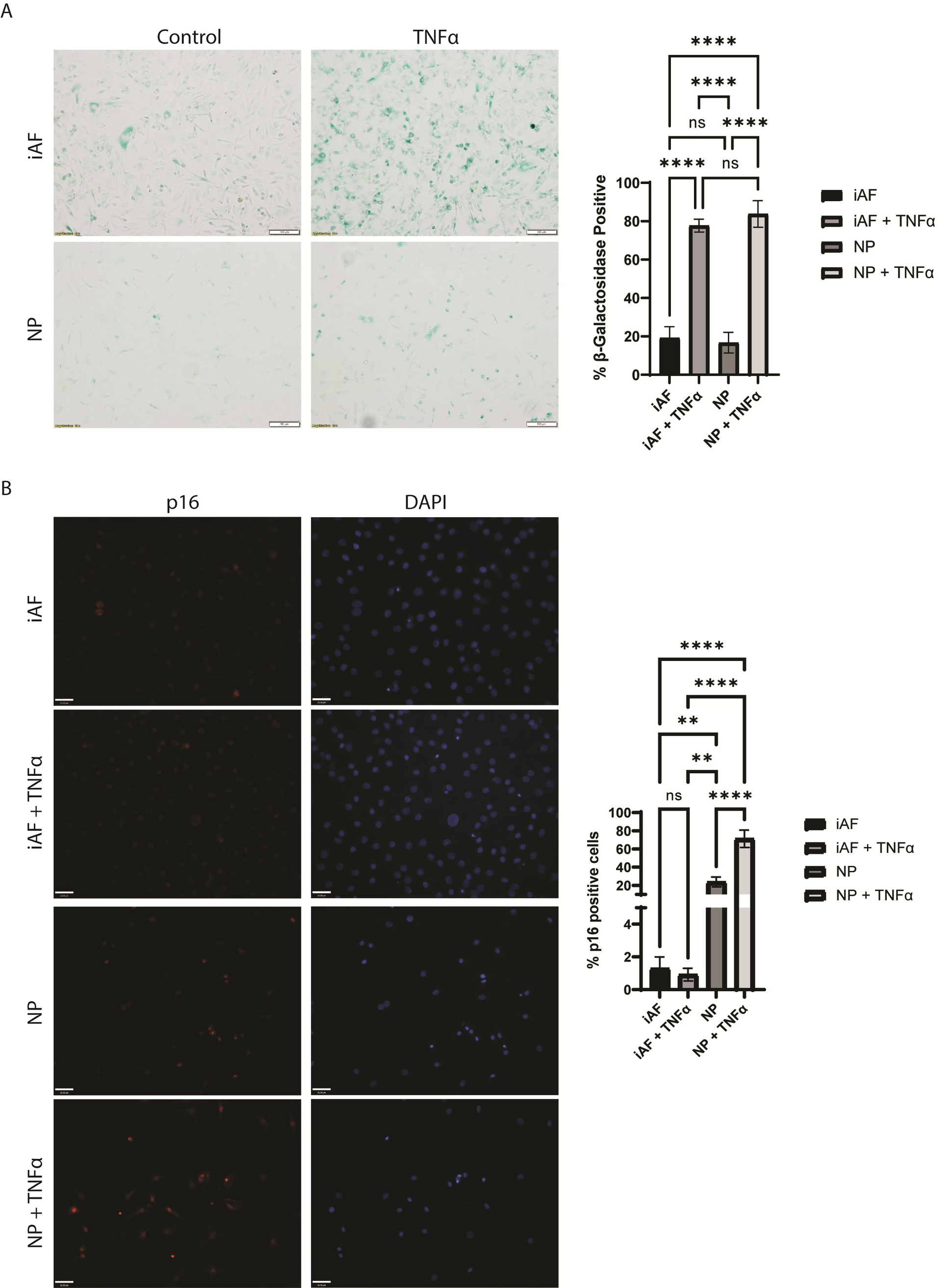

Supplement: S3 Fig — (A) Representative images and quantification of SA-βGal stained untreated controls and TNFα treated iAF and NP cells. (B) Representative images and quantification of p16 stained untreated controls and TNFα treated iAF and NP cells. p<0.05 = *, p<0.01 = **, p<0.001 = ***, p<0.0001 = ****, N = 3; Scale bar = 60 μm. (TIF) [file pone.0280101.s003.tif]

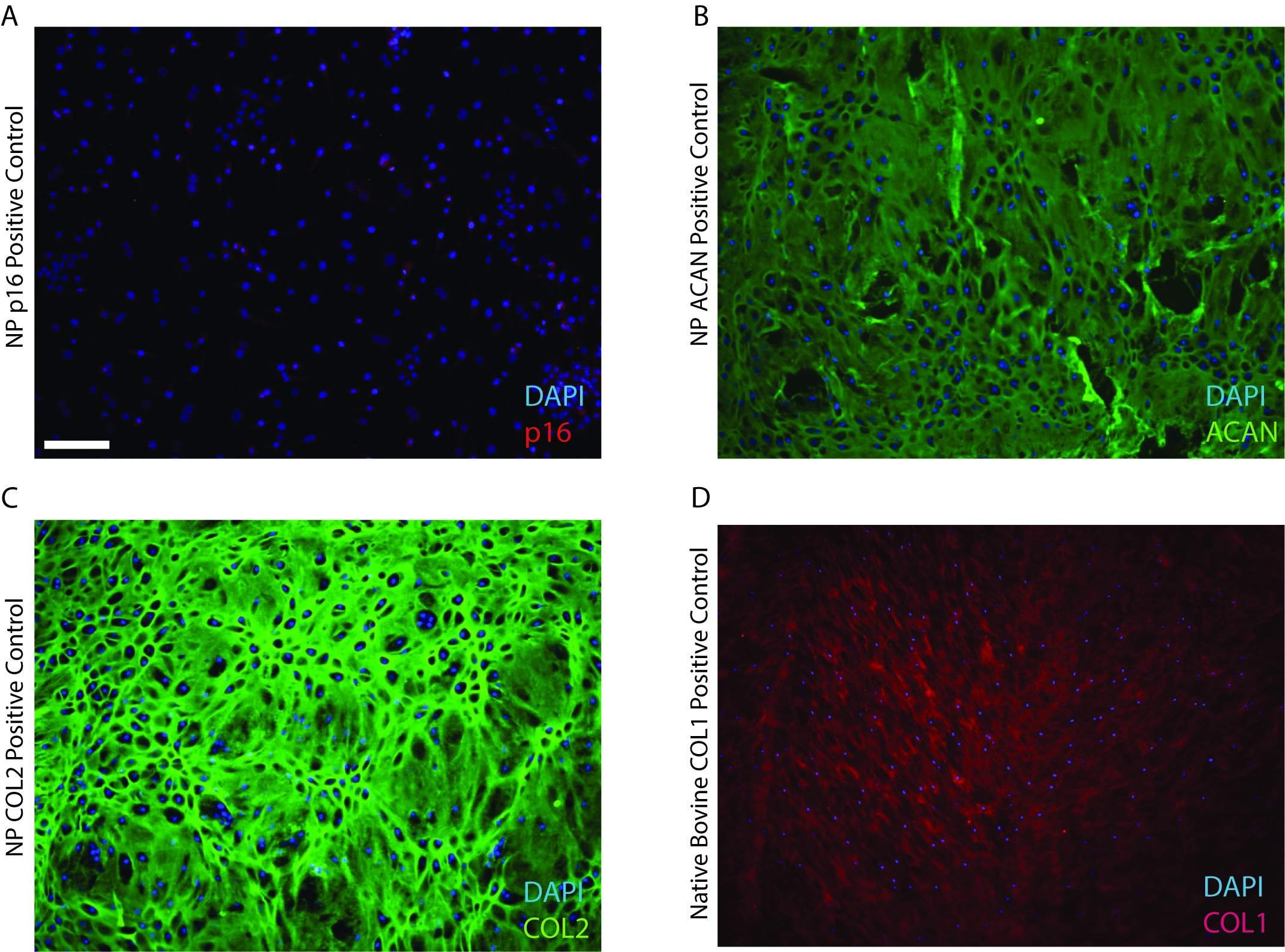

Supplement: S4 Fig — (B-C) Tissue formed by nucleus pulposus cells grown for 2 weeks in 3D culture stained for ACAN (B) and COL2 (C). (D) Native bovine IVD tissue stained with antibody reactive with COL1. DAPI was used as a nuclear counterstain. Scale bar = 100 μm. (TIF) [file pone.0280101.s004.tif]
