# Supplementary material for: Senescent response in inner annulus fibrosus cells in response to TNFα, H2O2, and TNFα-induced nucleus pulposus senescent secretome
Source: PLoS One. 2024 Jan 5;19(1):e0280101. doi: 10.1371/journal.pone.0280101 (PMC10769024; doi:10.1371/journal.pone.0280101)

## Supplemental 5: Methods

Western Blot and Densitometry

Protein samples were prepared by lysing NP and iAF cells at P1 with radioimmunoprecipitation assay (RIPA) lysis buffer with protease and phosphatase inhibitors. After quantifying the protein concentration with bicinchoninic acid assay (89900, Pierce, Waltham, MA, USA), samples were denatured and proteins separated by SDS-PAGE gel. The proteins were transferred onto a PVDF membrane, the membranes were then blocked using a solution of 5% skim milk (SK1400.500, BioShop, Burlington, ON, Canada) diluted in TRIS-buffered saline containing 0.1% Tween 20 (TBST). The membrane was incubated overnight at 4℃ with antibody reactive with TNF Receptor I (TNFR1) (1 μg/ml), followed by washes with TBST, and then incubation in secondary polyclonal IgG antibody (1μg/mL) linked to horseradish-peroxidase (HRP) at room temperature for one hour. The membrane was washed three times with TBST. Immunoreactivity was detected using Amersham ECL Prime Western Blotting Detection Reagent kit (RPN2235, GE Healthcare, Little Chalfont Buckinghamshire, UK). Protein bands were visualized using Bio-Rad ChemiDoc™ Imaging System. The membrane was stripped, blocked and incubated with GAPDH primary antibody (1 μg/ml) and then treated similarly as described above. The resulting bands were quantified using ImageJ 1.48v software (Wayne Rasband, National Institute of Health, USA). Three biological replicates were performed. The details of primary antibodies for TNFR1 and GAPDH is added into supplementary methods table 1, for the secondary antibody in supplementary methods table 2.

PCR
The primers are provided in supplementary methods table 3.

Table 1: List of primary antibodies.

| Primary Antibody | Species | Dilution | Company | Cat# | Antigen Retrieval |
| --- | --- | --- | --- | --- | --- |
| Type I collagen | Rb | 1:250 | Abcam | Ab34710 | Pepsin |
| Type II collagen | Ms | 1:250 | Millipore | MAB8887 | Pepsin |
| Aggrecan | Ms | 1:250 | Life Technologies | AHP002 | Hyaluronidase |
| MMP13 | Ms | 1:500 | GeneTex | GTX59793 | Dako pH9, 95˚C, 10 min |
| Cytokeratin (AE1/AE3) | Ms | 1:250 | Dako | GA053 | Pepsin |
| Ki67 (SP6) | Rb | 1:500 | ThermoFisher | MA5-14520 | None |
| p21 | Ms | 1:500 | ThermoFisher | PA1-30399 | None |
| p16 | Ms | Stock | Roche | 06695248001 | Monolayer: None  Tissues: Dako pH9, 95˚C, 10 min |
| TNFR1 | Rb | 1:1000 | Abcam | 19139 | N/A |
| GAPDH | Rb | 1:2000 | Millipore Sigma | ABS16 | N/A |

Table 2: List of secondary antibodies.

| Secondary Antibody | Species | Cat# |
| --- | --- | --- |
| AlexaFluor 594 | Goat anti-Rb | A32740 |
| AlexaFluor 594 | Goat anti-Ms | A32742 |
| AlexaFluor 488 | Goat anti-Rb | A32731 |
| AlexaFluor488 | Goat anti-Ms | A32723 |
| AlexaFluor 647 | Goat anti-Ms | A32728 |
| pAB IgG-HRP | Goat anit-Rb | ab6721 |

*All secondary antibodies are from ThermoFisher and used at a concentration of 1:500 in 20% goat serum, 0.1% Triton X-100, in PBS.

Table 3: List of primers.

| Gene | Forward primer (5’ to 3’) | Reverse primer (5’ to 3’) |
| --- | --- | --- |
| COL1A1 | CTACCACTGCAAGAACAGCGTG | CGTAGGTGACGCTGTAGGTGAA |
| COL2A1 | GTGTCAGGGCCAGGATGTC | GCAGAGGACAGTCCCAGTGT |
| ACAN | TGGGACTGAAGTTCTTGGAGA | GCGAGTTGTCATGGTCTGAA |
| IL6 | ATGATGAGTGTGAAAGCAGCAA | AGCAGTGGTTCTAATCAAGCAA |
| MMP13 | ATTGATGCCGCCTATGAGCA | AGGGCTGCGCTGATCTTTTT |
| SOD1 | GAATATTCCATCATTGGCCG | ATGAGTTACTAGGACTAAGCC |
| SOD2 | CTACGTGAACAACCTCAAC | AGAGCTATCTGAGCTGTAAC |
| CAT | CCTTTCTGTTGAAGATGCAG | ATGGCATTGAAAAGATCGC |
| NOX2/CYBB | CAAGAATTCGGAGACAACTG | TGAATTGCAGTGTGAAGTG |
| NOX4 | TCTGCATTAGAAAACCATCC | TACTATCTTAAGGTGGACCC |
| MKX | TCCTGTTCTGAAGATGGAG | ATTACGGGATGGTGAACTG |
| TNFR1 | TAACCACCCGCAAAATAGCA | GTTCTCCAAGGCAGTGTAGG |
| 18S | GTAACCCGTTGAACCCCATT | CCATCCAATCGGTAGTAGCG |

**Automated well overlay used to collect predetermined imaging sites. (A)** Image of an 18-well ibidi chamber with well-overlay highlighting the well (blue circle) and the potential imaging frames within the well (red squares with X’s). **(B)** Representative image of a single well with the well overlay. The white squares with arrows highlight the sites imaged in each well. The same 3 sites in each well were used for all monolayer imaging.

**A**


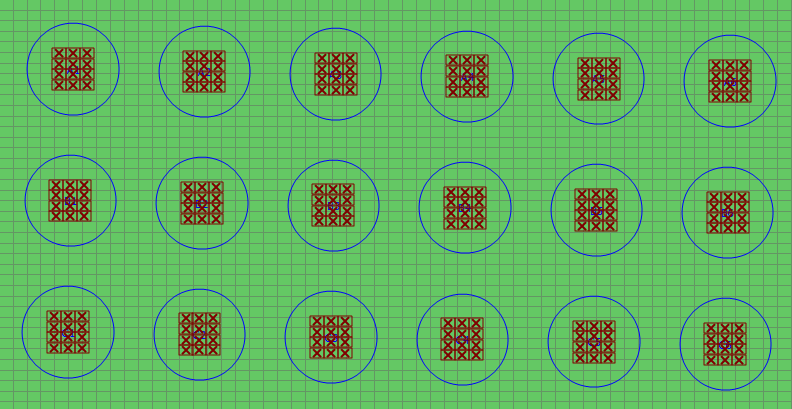


**B**


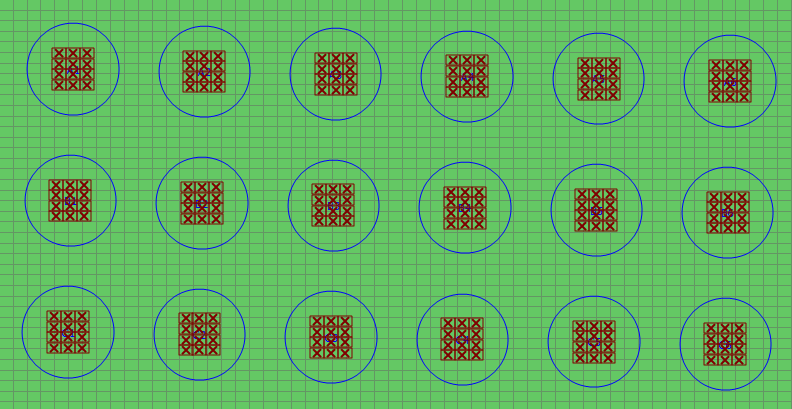

Supplement: S1 Methods — (DOCX) [file pone.0280101.s005.docx]
