# Supplementary material for: Senescent response in inner annulus fibrosus cells in response to TNFα, H2O2, and TNFα-induced nucleus pulposus senescent secretome
Source: PLoS One. 2024 Jan 5;19(1):e0280101. doi: 10.1371/journal.pone.0280101 (PMC10769024; doi:10.1371/journal.pone.0280101)
Supplement: Attachment — Submitted filename: Response to reviewer-submitted v2.docx [file pone.0280101.s006.docx]

PONE-D-22-34265

Senescent response in inner annulus fibrosus cells in response to TNFα, H_2_O_2_, and TNFα-induced nucleus pulposus senescent secretome
_____________________________________________________________________________

**Reviewer #1:**

The study “Senescent response in inner annulus fibrosus cells in response to TNFα, H_2_O_2_, and TNFα-induced nucleus pulposus senescent secretome” is evaluating an interesting and clinically relevant topic. It is proposing a novel difference between NO and iAF cells, but the data is not quite there to support the theory.

**General comments:**
**Comment 1-1**

*- The paper is aiming to compare NP and iAF cells but both cell types are often not included in the same set of experiments. Please correct.*

**Authors’ Action**

Thank you for your comment. We apologize for the confusion. Actually, the hypothesis of the study was not to compare iAF and NP cells, but rather to evaluate iAF cells under conditions which we know should induce senescence as we have shown for nucleus pulposus cells (Ashraf et al. 2021 doi: 10.1096/fj.202002201R). This former paper describes TNFα induced senescence in NP cells and explores mechanisms. Thus, we did not include those experiments/results, but instead we provided the reference of this previously published paper in the revised version of this manuscript.

We do acknowledge that we made some comparisons between the response of NP and iAF cells to TNFα, however, this was only to provide the support for our rationale for exploring a potential mechanism for this differential senescence sensitivity and also to provide context for the role of ROS/H_2_O_2_ regulation within the iAF. We have modified the abstract to state this. Also we have re-ordered these descriptions and modified the results on page 16, starting on line 327 which we hope makes it clearer that these comparisons to NP are provided only to confirm they are responding differently (and also to serve as a positive control to demonstrate the reagents were effective).

However, we did repeat the SA-βGal and p16 staining experiments using NP and iAF cells obtained from the same disc harvests as requested. The staining was done at the same time and show similar results to the original experiments (data shown Supplemental Figure 3).

**Comment 1-2**

*-Please change the title to reflect the study.*

**Authors’ Action**

We hope that with the rewrite of the results that it is clearer that the NP studies are present to only support the differential response of the iAF. The original title focuses on the study which is the characterization of iAF cells – the main aim of the study. As some of the NP results are in another paper, we would prefer to keep the title the same (Senescent response in inner annulus fibrosus cells in response to TNFα, H_2_O_2_, and TNFα – induced nucleus pulposus cell senescent secretome). However, we are willing to change it if the reviewers feel it is better to include NP in the title.

**Comment 1-3**

*-The references are outdated and don’t reflect the current state of the art. Please update.*

**Authors’ Action**

Thank you for your comment, we apologize for this. Where appropriate older references have been replaced by more recent publications and the reference list revised to incorporate them and deletion of older papers.

**Comment 1-4**

*-A positive control for each AB should be included in each experiment for confirmation.*

**Authors’ Action**

Positive immunostaining control images have been added as supplemental figure 4 and referenced on Page 16, line 324 and page 20, line 433.

A legend was added that describes this as follows: **“**Immunohistochemical positive control images of (A) NP cells stained with antibody reactive with p16 following 24hr exposure to TNFα. (B-C) Tissue formed by nucleus pulposus cells grown for 2 weeks in 3D culture stained for ACAN (B) and COL2 (C). (D) Native bovine IVD tissue stained with antibody reactive with COL1. DAPI was used as a nuclear counterstain**.”**

**Comment 1-5**

*-In general, the IHC are of low quality and are not reflecting the quantifications. Please correct.*

**Authors’ Action**

Control matched linear adjustment of brightness and contrast was made to figure images, where necessary, to improve visibility of scaled down immunofluorescent images. We hope you can see them more readily.

**Comment 1-6**

-*The figure legends in the text are mislabeled. For example, there are 3 figure legends labelled Fig 1. Please correct.*

**Authors’ Action**

We apologize for this error and the legends have been renumbered to correct this.

**Comment 1-7**

*-The supplementary figure S2 is not mentioned in the text.*

**Authors’ Action**

Thank you for picking this up. We have now referenced Supplemental Figure 2 in the text to highlight that iAF cells treated with TNFα or H_2_O_2_ did not increase cell death or apoptosis and that iAF or NP cells treated with DPI did not increase cell death. (Page 17, lines 348-350 and page 19, line 398).

**Comment 1-8**

*-The number of biological replicates seems too low especially since there is significant variability between the 3 biological replicates*.

**Authors’ Action**

All independent experiments were performed at least three times (3 biological replicates) using at least three different caudal spines and each independent experiment had three technical replicates unless otherwise specified. We believe in the original figures, the variability between the biological replicates may have looked exaggerated because the y-axis range was too small. We have corrected the y-axis range in some of the figures to standardize the y-axis range across experiments where it was appropriate and did not cause loss of information. We have also included an acknowledgement of the possible sample size limitation in the discussion. (Page 28, lines 607-609)

**Specific questions,**

**Comment 1-9**

*Figure 1. It is clear from the literature that SA-βGal is overestimating and p16 underestimates the number of senescent cells, but I have never seen a disconnect of this magnitude. It would help interpret the data if NP and iAF cells were treated and analyzed simultaneously.*

**Authors’ Action**

Thank you for your comment. As we described in our response to comment 1-1,

we repeated the SA-βGal and p16 staining experiments on NP and iAF cells from the same population (same cell isolation) at the same time. Our results remain consistent to the original experiments. This data is show in Supplemental Figure 3. A legend was also added in on page 55.

**Comment 1-10**

*Figure 1, B, please provide the same analysis for NP cells. The cells that escaped senescence would continue to proliferate and comparing the two cell types would indicate if the presence of senescent cells affects the continued proliferation.*

**Authors Action 1-10**

Thank you for your comment. As mentioned above, the aim of the study was not to compare iAF and NP cells, but rather to evaluate the conditions in which iAF cells undergo senescence. We hope with the changes to the text of the manuscript this is now clearer.

Evaluating percent Ki67 positive cells was done as a second way to confirm that TNFα was not inducing growth arrest in iAF cells, which is used as another marker of senescence. Quantifying Ki67 cells has been shown to be a good way to assess this (Lawless C. et al. 2010 doi: 10.1016/j.exger.2010.01.018). The conclusion was supported by doing a growth curve to show cell number is increasing in these cells. If senescence was occurring, a reduction in the number of cells relative to untreated control would be expected if a substantial fraction of the total cell population underwent senescence. The positive control (serum starvation) showed growth arrest can be detected with this approach.

**Comment 1-11**

*E, F, there are no visible p16-positive cells, how can about 10% detected? Please provide images that reflect the quantification.*

**Authors’ Action**

We apologize for this. Control matched linear adjustment of brightness and contrast was made to figure images, where necessary, to improve visibility of scaled down immunofluorescent images.

**Comment 1-12**

*Figure 2. The y-axis should have the same scale across the analysis to allow for comparison.
B and E. The iAF data is much more variable than NP and would need more biological repetitions to give solid data.*

**Authors’ Action**

As we described in our response to 1-8, we have adjusted the y-axis across all of the graphs in the study to be consistent when comparing the same gene/assay. As mentioned above, we have also included an acknowledgment of the sample size limitation in the discussion. (Page 28, lines 607-609).

**Comment 1-13**

*I detail but it seems that the image in 2D is not the same for Dapi and CellROX*

**Authors’ Action**

Thank you for your comment. We apologize for the confusion. The image in figure 2D is the same, however, it was mistakenly flipped. We have corrected this in the revised manuscript.

**Comment 1-14**

*G-H The data is too variable to make the proposed conclusions. PCR data usually requires a minimum of n=6 to give solid data. Was a power analysis performed? I would also suggest presenting the data normalized to its own untreated control (2–∆∆Ct). Please mention the ct values of the housekeeping gene under the different treatments. Please add more biological replicates.*

**Authors’ Action**

Thank you for your comment. In this study we did not use a power analysis as most gene expression data was evaluated using at least 3 independent biological replicates, each having 3 technical replicates (total of 9 samples per experimental condition). We have used this in our previous studies and this number was sufficient to detect differences. However we have added this in as a potential limitation of our study in the discussion on (Page 28, lines 607).

With regards to the 2^–∆∆Ct^, we chose to use 2^-∆CT^ over 2^-∆∆CT^ as we believe this more accurately reflects variance within the control and treatment conditions. When using 2^-∆∆CT^ all control samples are set to 1 which makes the standard deviation of the control condition 0. We think this can be misleading when examining only 2 conditions.

We have provided the average±SD of 18s rRNA Ct values in the figure legends where appropriate. (Page 17, lines 359-362; page 19, lines 409-410; page 20, lines 424-429; page 22, line 469)

**Comment 1-15**

*Figure 3. Please show data for NP and iAF cells to facilitate comparison and validate the conclusion.*

**Authors’ Action**

The main aim of this figure was to evaluate the senescence response of iAF cells exposed to H_2_O_2_ so adding in NP data does not advance this. As described in the response to comment 1-1 we were not evaluating NP response to H_2_O_2._ We hope with the modifications to the manuscript described above that this is now clear. While future work could explore the differential response in greater detail, we first looked to characterize the iAF response to physiologic stimuli thought to contribute to senescence within the IVD.

**Comment 1-16**

*B-C please show images reflective of the quantification.*

**Authors’ Action**

We apologize for this. Control matched linear adjustment of brightness and contrast was made to figure images, where necessary, to improve visibility of scaled down immunofluorescent images.

**Comment 1-17**

*Figure 4A, Please see comments in 2G-H*

**Authors’ Action**

Please see authors’ action 1-14.

**Comment 1-18**

*Figure 5. Please show data for NP and iAF cells to facilitate comparison and validate the conclusion.*

**Authors’ Action**

Thank you for your comment. As mentioned above, the aim of the study was not to directly compare iAF and NP cells. We have previously published on NP cell senescence and the impact on tissue formation (Ashraf et al. 2021 doi: 10.1096/fj.202002201R and Ashraf S et al 2020 doi: 10.1002/jor.24476).

**Comment 1-19**

*Please adjust the discussion in light of the additional experiments.*

**Authors Action 1-19**

Thank you for your comment. We have made changes to the discussion to present some limitations that were highlighted by the reviewers. Although additional experiments were done and added to the supplemental figures, they did not substantially change the conclusions.

**Reviewer #2:**

**Comment 2-1**

*My suggestions including a detail timeline of the NP and iAF cultures, iAF and NP cell characterization at the expression level, curves of Dose- effect of H2O2 and TNF specific for iAF and NP, time effect to recognize the ability of the iAF and NP cells to respond to the stressors (TNFα, H2O2).*

**Authors’ Action**

Thank you for your comment. The dose of TNFα was selected based on our previous work which showed that NP cells underwent senescence at that concentration (40ng/mL). A time course was not done in this study as we wanted to determine if iAF cells responded similarly to TNFα at a concentration known to induce senescence in other cell types. We have added this to the text to make this clearer on page 6, lines 115-116.

**Comment 2-2**

*Unfortunately, immunostaining and in general images doesn’t reflect the quantification results. I strongly suggest high magnification in the images and inclusion of a complementary iAF markers.*

**Authors’ Action**

We apologize for this. Control matched linear adjustment of brightness and contrast was made to figure images, where necessary, to improve visibility of scaled down immunofluorescent images.

Unfortunately, there is no marker specific to iAF cells which is why we are unable to do this evaluation. iAF cells express both collagen type II and type I, whereas outer AF expresses only type I collagen. We use the collagen types as surrogate biomarkers for iAF cells.

**Comment 2-3**

*Figure 1*

*• SA- galactosidase staining images show only one area of the plate. It is important to show if all the analyzed samples/plates are positive to galactosidase at the same levels.*

**Authors’ Action**

Thank you for your comment. We have included a low magnification image of the entire culture stained with SA-βGal (Figure 1A). Although we show a representative high power image, it should be noted, we used a standardized method of counting cells in all our cultures for SA-βGal that eliminates selection bias of areas to count. This methodology is described in detail in Supplementary figure 5. We modified the description in the methods section on page 11, lines 222-223 as follows:

“The images were captured from the same location in each well using a standardized well-overlay method (described in S5 methods).”

**Comment 2-4**

*• The number of cell/well (Figure 1B) do not correlate with the % of the Ki67 positive cells. The trend of %Ki67 shows decreasing in cell proliferation with time (72hrs) in all the conditions. Representative images do not show a clear Ki67 staining to demonstrate differences/similarities between groups. Are the galactosidase and Ki67 positive cells distinct populations?*

**Authors’ Action**

Thank you for your comment. Ki67 stains proliferating cells from G1 through M. We likely have cells in all stages of the cell cycle so the percentage of Ki 67 positive cells may not exactly correlate with the cell number observed in the growth curve. Secondly, Ki67 does not stain all proliferating cells equally, as protein levels can drop during G0/1 for example. Thus, the levels of Ki67 can be heterogeneous (Miller I, et al. 2018 doi: 10.1016/j.celrep.2018.06.110.). The authors also show “Ki67 levels fall as a function of time since quiescence entry, quiescent cells re-entering the cell cycle will have varying levels of Ki67 depending on how long the cells have been quiescent”. The positivity threshold we used to determine a positive (vs negative) stained cell may have decreased the sensitivity of this evaluation and under-represented total proliferating cells. However, this threshold was the same for all treatment conditions evaluated. We would argue that comparing percent Ki67 positivity and growth curves directly may not be appropriate given the experimental and biological constraints.

With regards to the decreasing Ki67 positivity over time, this was expected because we did not change the media over the 72hrs following TNFα removal. This was done as previous studies have shown that the secretome of senescence cells can signal in an autocrine and paracrine manner to induce further senescent changes. In this way, we hypothesized not changing the media would give the greatest likelihood of observing senescence effects in the TNFα treated iAF cells – which we still did not observe. We have now added in a statement stating that the media was not changed during the evaluation of the growth curves to clarify this on page 10, line 209-210.

With regards to distinct cell populations, cells used for the Ki67 evaluation are fixed differently (paraformaldehyde) than cells stained for SA-βGal (2% formaldehyde/0.2% glutaraldehyde) so it is not possible to co-stain cells and determine if these positive cells are distinct populations. To try to address this we did a growth curve to show that cell number was increasing which supports the finding that TNFα treatment is not inducing senescence in iAF cells. Although this does not exactly address your question it does provide additional information and is used by others to evaluate for the presence/absence of senescence (Gonzalez-Gualda E. et al. 2021 doi: 10.1111/febs.15570).

**Comment 2-5**

*Figure 2*

*• Fold change in the SOD1 SOD2 and CAT, NOX2 and NOX4 gene are slightly different between the experimental and control treatments in NP vs iAF. Then, it is difficult attribute differences in the ROS response of the NP and Iaf*

**Authors’ Action**

Thank you for your comment. The data is not expressed as fold change. It is expressed as 2^-∆CT^ as we believe this better shows the variances within the control and treatment conditions. When using 2^-∆∆CT^ control samples are set to 1 which makes the standard deviation of the control condition 0. This can make fold change data less suitable for statistical evaluation in a two condition experiment.

With respect to the differential expression we observe between the NP and iAF cells in the untreated control condition, this was expected. NP and iAF are distinct cell populations derived from different embryonic lineages with unique transcriptomes. Previous studies have highlighted a differential expression of genes between untreated iAF and NP cells such as for SOD2 [Kudelko, M et al. 2021 doi: 10.1016/j.mbplus.2021.100082, Panebianco, CJ et al. 2021 doi: 10.1096/fj.202101149R]. As untreated iAF and NP cells had differential gene expression, that is why we analyzed their respective transcriptional response to TNFα independently and comparison was to their respective control cells only. Thus, it is possible to detect differences in ROS response in NP or iAF cells.

**Comment 2-6**

Figure 3

*• Line 360. Figure legend should be Figure 3 instead of Figure1*

**Authors’ Action**

Thank you for picking this up and apologize for the typo. This has been corrected in the revised version of the manuscript.

**Comment 2-7**

*• Images of P16 and P21 immunostaining do not show positive staining or is difficult to see them. I strongly suggest images at 10x and 20x magnification then H2O2 changes could be visualized.*

**Authors’ Action**

We apologize for this. Control matched linear adjustment of brightness and contrast was made to figure images, where necessary, to improve visibility of scaled down immunofluorescent images. We hope with this change, immunostaining can be seen.

**Comment 2-8**

*• Line 378. Figure legend should be Figure 4 instead of Figure2.*

**Authors’ Action**

Thank you for picking this up and apologize for the typo. This has been corrected in the revised version of the manuscript.

**Comment 2-9**

*• Cell quantification of Col1, Col2 and Aggrecan immunostaining and images do not show the same data. It is not clear what is the staining vs background levels. Immunostaining must reflect the quantification data from the graphs (Figure 3)*

**Authors’ Action**

Thank you for your comment. We have increased the brightness and contrast of these images to improve visibility in the revised manuscript. We think that the images and quantification do correlate, as the % positive cells shown in the images fall within the range displayed in the graphical data. For example, in the quantification of COL2 (Fig4B) the control sample in the image displayed has 45.4% positive cells, where the TNFα treated condition has 21.2% positive cells. This is within the range of plotted results on the associated graph (Control: 33-46%, TNFα: 15-25%).

**Comment 2-10**

*• Line 399. Figure legend should be Figure 5 instead of Figure3.*

**Authors’ Action**

Thank you for picking this up and apologize for the typo. This has been corrected in the revised version of the manuscript.

**Comment 2-11**

*• I highly recommend have a live/dead assay as support data to be certain about the percentage of live cells and the successful 3D cultures.*

**Authors’ Action**

Thank you for your comment. We had performed Live/Dead assays and TUNEL staining on TNFα and H_2_O_2_ treated iAF cells (Supplemental figure 2). In these studies, none of the treatment conditions were found to be cytotoxic. We did not include in-text citations for this figure of the original manuscript which we corrected in the revised version of the manuscript. (Page 17, lines 348-350 and Page 19, lines 396-398).

**Comment 2-12**

• *Line 421. Figure legend should be Figure 6 instead of Figure 4.*

**Authors Action**

Thank you for picking this up and apologize for the typo. This has been corrected in the revised version of the manuscript.

**Comment 2-13**

*• Line 427. Figure legend p21 instead of p12*

**Authors Action 2-13**

Thank you for picking this up and apologize for the typo. This has been corrected in the revised version of the manuscript (now on page 19, line 408).
