# Supplementary material for: Senescent response in inner annulus fibrosus cells in response to TNFα, H2O2, and TNFα-induced nucleus pulposus senescent secretome
Source: PLoS One. 2024 Jan 5;19(1):e0280101. doi: 10.1371/journal.pone.0280101 (PMC10769024; doi:10.1371/journal.pone.0280101)
Supplement: Attachment — Submitted filename: ReviewerResponse_resub2_AMS2-rk.docx [file pone.0280101.s007.docx]

**PONE-D-22-34265R1**

Senescent response in inner annulus fibrosus cells in response to TNFα, H2O2, and the nucleus pulposus SASP secretome

PLOS ONE
_____________________________________________________________________________
**Academic Editor:**

1) As Reviewer 1 pointed out in their first review, a n = 3 might be too low to draw solid conclusions about the effect of treatments, especially when considering the minor (while significant) changes between groups. Please acknowledge this limitation in greater detail in the discussion.

- *Thank you for your comment. We have expanded our discussion of this limitation including “Given that some of the effect sizes, although significant, were limited, the biologic relevance of these findings will require further investigation.” (lines 604-605)*

2) In addition, as reviewer 1 requests, please add data about TNFaR expression to the manuscript.

- *Thank you for your comment. We have analyzed for TNFR1 gene and protein expression in iAF and NP. The data is added into the manuscript as supplemental figure 5. We have also described the findings in the abstract and results “Additionally, gene and protein expression show that TNFR1 is expressed by both NP and iAF cells, however, TNFR1 protein expression is significantly higher in iAF cells than NP cells (Fig. S5).” (lines 368-370). The discussion was modified to incorporate this information. For example we added to the discussion “Furthermore, both iAF and NP cells had measurable levels of TNFR1 gene and protein expression, which suggests the differential senescence and degeneration response to TNFα is likely downstream of the TNFR1.” (lines 561-563). We have included the details of the antibodies and primers used in tables 1 - 3. As well the western blot method and densitometry description has been added into the supplemental methods figure now labelled Supplemental Figure 6 Methods.*

3)Regarding Figure 6: why has 6 B 6 data points for quantification? The figure legend states that it was n=3. Was the statistics performed with n=3 or n=6? Please correct the figure and perform statistics for n=3.

- *We apologize for this error. We have clarified in the legend of figure 6 that N=6 for collagen and aggrecan immunostaining (line 477). The statistics were all done with the appropriate replicates*.

**Reviewer #1:**

The manuscript is significantly improved but a few concerns remain.

**Comment 1-1**

Dr. Lemaitre showed in 2007 that cells in non-degenerate IVD tissue have very low TNFAR expression (Arthritis Research & Therapy 2007, 9:R77 (doi:10.1186/ar2275). Based on this it would be important to determine if bovine NP and iAF cells express the same level of the receptor. This knowledge would confirm if the results were due to the availability of the receptor or if the downstream signalling differs. Please add data and discussion.

- *Thank you for your comment. We have added TNFR1 gene and protein expression data to supplemental figure 5 and have commented on these data in the results and discussion (see academic editor comment 2).*

*Interestingly, iAF cells had higher protein expression of TNFR1 than NP cells (despite similar gene expression). This suggests either that the differential response in iAF vs NP cells to TNFα is due to (1) the differential TNFR1 concentrations induce a differential response (although we believe this is unlikely given NP cells undergo senescence when exposed to TNFα despite low TNFR1 protein expression), (2) that the differential response is due to downstream factors that influence TNFR1 response(s), or (3) that the differential response may be due to activation of other TNFα superfamily receptors (a family that comprises 27 different receptors), or possibly (4) a combination of the above.*

**Comment 1-2**

Figure 3 E control is flipped.

- *Thank you for detecting this. We have addressed this issue in the revised version of the manuscript.*

**Reviewer #2:**

The study Senescent response in inner annulus fibrosus cells in response to TNFa, H202, and TNFa-induced nucleus pulposus senescent secretome. The study proposes NP and inner AF differences particularly in the way to respond to TNFa, H2O2 and in the cell coculture by influence on NP-TNF treated cells. The proposing idea is novel and interesting for the Disc community. My questions-concerns were address in the proper way, however the manuscript still require a little bit of improvement at the edition level.

**Comment 2-1**

Line 1-37. I highly recommend include the socioeconomic impact of the back pain and DDD at the worldwide, because this is one of the most prevalent issues at global level.

- *Thank you for your comment. We have added a statement to the introduction discussing the global impact of IVD degeneration/back pain beyond just Canada. (lines 40-41). A reference was added to support this statement and all the subsequent references were renumbered.*

**Comment 2-2**

46-47 and 81-82. Sentence “many to believe” Is better to write a fact than write an general opinion.

- *Thank you for your comment. We have changed the wording where applicable.*

**Comment 2-3**

Methods section

Be homogeneous in the way of including the company, country of origin and use reagents, e.g. sometimes is general: line 94, “Winset, 318-010-cl” ,line 101 “Winset products” sometimes is very specific: line 95 “protease (Type XIV, P5146 Sigma-Aldrich, St. Louis, MO, USA), sometimes even is not included the company name e.g. FBS, line 104. Please correct in all methodology.

- *Thank you for your comment. We have changed all product references to be consistent ("product number, company name, city and country”, ) where applicable.*

**Comment 2-4**

Line 99: even if the digestion timing was variable is not the same 16 hrs than 10hrs or 20hrs so please be most precise with the incubation times, if someone try to reproduce your experiments, issues as over digestion or poor tissue dissociation can affect enormously the results.

- *Thank you for your comment. We have changed the wording in “Cell isolation and monolayer culture to reflect the variance of the digestion time, which was 16hrs ±1hr. (line 107).*

**Comment 2-5**

Line 102, 105, 196, 221, 238, 249: same situation than before. I assume that cell counting was performed by manual or automated counting so by precisely with the number of cells.

- *Thank you for your comment. We have added a line in the methods describing the manual cell counting was used (line 128 and 130) and removed “approximately” from all cell counting numbers.*

**Comment 2-6**

Line 145: Formation of the tissue sheet. It was not described what kind of supportive material was used to seed the iAF. Authors can be precise if was home made or sheets were from specific company and how the 3D structures were performed.

- *We apologize for confusion around this method. We formed the cell sheets exactly as described in the methods. Cells were seeded directly on uncoated polystyrene culture plates. Cells were seeded at high density, grown in the complete media described in the manuscript, and cultured for 10 days to accumulate the matrix molecules they produce as extracellular matrix and form a 3D tissue. We have modified the method starting on line 162 to try to clarify this. On lines 173-174 the following statement was added-* Under these culture conditions, the cells accumulate extracellular matrix molecules they synthesize and form tissue.
- *However we can see how it can be confusing to say we grew the tissue in 3D when actually it is the high density culture that resulted in tissue formation which is 3D. We have changed this in the results section on lines 437-438 so it now states-* cells were also grown in high density culture so they formed tissue.

*We also removed 3D and replaced it with tissue where appropriate, eg line 440 3D was changed to* tissue.

**Comment 2-7**

Line 296: Figure 1. Unfortunately, it was not uploaded the new Figure 1 instead is the figure 2 in the attachments. In the pdf the resolution is very poor, I would like to the high quality image.

- *We were able to identify why the figures were being mislabeled and this has now been fixed so the appropriate figure 1 is provided. We apologize for the confusion.*

**Comment 2-8**

Line 329-330: Figure 2: Although in the text it was mentioned the AF and NP comparison. Is difficult to interpret the figure. Is unclear which data/graphs correspond to iAF and which to NP. Maybe subtitles or frames-grouping can help to understand better the figure.

- *Thank you for your comment. We have added additional labels to subfigures 2 G and H to help distinguish iAF vs NP data. Subfigures I and J have titles above the graphs to highlight NP vs iAF.*

**Comment 2-9**

Figure 3,4: Same than above make clear NP and iAF graphs.

- *Figure 3 and 4 only present iAF data.*

**Comment 2-10**

S4: Figure S4 need the figure legend or subtitles on top of images. They are only label with A,B,C,D.

- *Thank you for your comment. We have included labels to supplemental figure 4 indicating the tissue used and the antibody used.*

**Comment 2-11**

Discussion section: The discussion section was deep enough, covering different perspectives of the topic and the body of information is sufficient to support the data depicted in the manuscript.

- *Thank you for all of your comments.*
