# Supplementary material for: Senescent response in inner annulus fibrosus cells in response to TNFα, H2O2, and TNFα-induced nucleus pulposus senescent secretome
Source: PLoS One. 2024 Jan 5;19(1):e0280101. doi: 10.1371/journal.pone.0280101 (PMC10769024; doi:10.1371/journal.pone.0280101)
Supplement: Attachment — Submitted filename: Response to reviewers 3.docx [file pone.0280101.s008.docx]

**PONE-D-22-34265R2**

**Senescent response in inner annulus fibrosus cells in response to TNFα, H2O2, and TNFα-induced nucleus pulposus senescent secretome.**

**PLOS ONE**

Dear Dr. Illien-Jünger,

Thank you for reviewing our manuscript (PONE-D-22-34265R2) and allowing us the opportunity to respond to the reviewer’s comments. I will note we have made some grammatical changes that we identified as we re-read the manuscript. These are tracked as well.

**Reviewer #1:**

Thanks for providing additional data. It is very clear from the additional data that TNF receptor expression at the protein level is many times lower in NP than AF cells. This is not clearly acknowledged. The response to TNF will likely be much milder in NP cells (and potentially resulting in a different secretory phenotype) which would explain why senescence is not induced to the same extent as in AF cells. Activating the cells by for example IL1 might equally induce senescence in both cell types as is shown for induction with peroxide. The difference is therefore likely based on receptor availability rather than different responses. Unless the secretory phenotype is verified at the protein level together with an evaluation of the downstream signalling, it must be clearly stated in the discussion and abstract that the difference may be due to receptor availability which could result in differences (or a much weaker response) of the secretory phenotype. Figure S5 is critical for the discussion and should included in the main text of the manuscript and not as a supplement figure.

Author’s Response:

We appreciate your comments. We acknowledge the substantial difference in TNF-α receptor expression at the protein level between NP and AF cells, which may indeed underlie the variations in senescence induction and the secretory phenotype observed between the two cell types.

We have addressed this point in the results section, emphasizing the magnitude of this difference (Lines 373-377). We have moved Figure S5 into the text and is now Figure 3. The subsequent figures have been renumbered and modified in the manuscript as appropriate.

We have added into the discussion the concept of receptor availability possibly driving the observed differences, and also included a statement about the need for further experiments to verify these hypotheses at the downstream signaling ( Lines 534-536; Lines 560- 563)

We modified the Abstract to reflect the presence of TNFR1 in iAF cells (Line 21-22).

Top of Form

**Reviewer #2:**

Authors properly addressed the previous comments. A significant improvement of the manuscript was done. I do not have further comments.

Author’s Response:

Thank you very much for helping us improve our manuscript.

We hope with these changes the manuscript is now acceptable for publication in PLOS ONE. Should you require any further information please do not hesitate to contact me.

Yours Sincerely,

Rita Kandel
